# Genetic analysis of a quantitative trait locus associated with resistance to the root-lesion nematode *Pratylenchus neglectus* in triticale

**DOI:** 10.1007/s00122-025-05112-6

**Published:** 2026-01-05

**Authors:** Gurminder Singh, Krishna Acharya, Bonventure Mumia, Siddant Ranabhat, Ekta Ojha, Jatinder Singh, Upinder Gill, Sean Walkowiak, Harmeet Singh Chawla, Xuehui Li, Justin Faris, Zhaohui Liu, Guiping Yan

**Affiliations:** 1 Department of Plant Pathology, North Dakota State University, Fargo, ND 58102 USA; 2 Department of Plant Sciences, North Dakota State University, Fargo, ND 58102 USA; 3 Grain Research Laboratory, Canadian Grain Commission, Winnipeg, MB R3C 3G8 Canada; 4 Department of Plant Science, University of Manitoba, Winnipeg, MB R3T 2N2 Canada; 5 USDA-Agricultural Research Service, Cereal Crops Research Improvement Unit, Edward T. Schafer Agricultural Research Center, Fargo, ND 58102 USA

## Abstract

Message.

A QTL from rye chromosome 5R confers resistance to root-lesion nematode in triticale.

Root-lesion nematode (*Pratylenchus neglectus*, RLN) poses a significant threat to global wheat production. High levels of RLN resistance are rare in wheat. Triticale, an amphiploid generated by combining wheat and rye genomes that naturally carries rye-derived defense alleles, offers an untapped reservoir of nematode resistance. Here, we evaluated the response to RLN in 137 recombinant inbred lines (RILs) derived from a cross between two triticale cultivars: Siskiyou (susceptible) and Villax St. Jose (resistant). Genotyping-by-sequencing identified 1054 high-quality single-nucleotide polymorphism (SNP) markers, which, along with seven simple sequence repeat (SSR) markers, were assembled into 21 linkage groups covering the triticale genome. A single quantitative trait locus (QTL) on the rye-derived chromosome 5R was identified that explained approximately 20% of the phenotypic variance across experiments. A high-throughput Kompetitive allele-specific PCR (KASP) assay based on the most significant SNP marker was developed, providing a rapid genotyping platform for selecting the resistance allele and reducing reliance on labor-intensive phenotyping for *P. neglectus* resistance in triticale. This study reports the first mapped RLN-resistance QTL in triticale, laying the fundamental foundation for introgressing the 5R resistance allele into wheat via marker-assisted selection combined with chromosome engineering, thereby broadening the genetic basis for nematode resistance in cereal crops.

**Supplementary Information:**

The online version contains supplementary material available at 10.1007/s00122-025-05112-6.

## Introduction

Root-lesion nematodes (RLNs; *Pratylenchus* spp.) are migratory endoparasites that attack plant roots and are recognized as widespread pathogens of wheat and other small grains worldwide (Castillo and Vovlas 2007; Smiley and Nicol 2009). These nematodes invade and cause lesions in root tissues, impairing water and nutrient uptake, and can cause substantial yield losses in infested fields (Smiley 2021). In rainfed wheat-growing regions, *P. neglectus* has been reported to reduce grain yields by up to 30% in Australia and 37% in the USA (Vanstone et al. 2008; Smiley and Machado 2009). Along with *P. thornei*, *P. neglectus* is among the most prevalent RLN species in temperate cereal production zones (Smiley and Nicol 2009), posing a significant economic threat to global cereal production.

Effective management of RLNs in the field is notoriously challenging (Vanstone et al. 2008; Dababat et al. 2016). Crop rotation with non-host or poor-host species can suppress nematode populations, but this strategy is constrained by the broad host range of RLNs and the need for profitable rotation crops (Smiley 2021). In many cereal-based production systems worldwide, common rotation crops such as barley, corn, soybean, and field pea can still serve as hosts for *P. neglectus*, thereby limiting the long-term efficacy of crop rotation as a control strategy (Vanstone et al. 2008; May et al. 2016; Thompson et al. 2016; Mokrini et al. 2019). Therefore, deploying host resistance is viewed as the most economical and sustainable approach for managing *P. neglectus* in cereal-based production systems (Smiley and Nicol 2009; Smiley 2021; Singh et al. 2023). Resistant cultivars can suppress nematode reproduction, reducing population densities to below economic injury level and mitigating damage over time (Cook and Evans 1987).

Extensive phenotypic surveys have revealed that high-level resistance to *P. neglectus* is rare in bread wheat (Vanstone et al. 1998; Taylor et al. 1999; Mulki et al. 2013; Dababat et al. 2016; Singh et al. 2023). Most modern cultivars are susceptible hosts, and even tolerant lines permit substantial nematode multiplication, perpetuating soil inoculum (Thompson et al. 2016; Singh et al. 2023). Genetic analyses indicate that resistance in wheat is typically quantitative, controlled by multiple loci of small to moderate effect (Zwart et al. 2010; Dababat et al. 2016). To date, only one locus of major effect, *Rlnn1* on chromosome 7AL, has been identified (Williams et al. 2002; Jayatilake et al. 2013). Although the *Rlnn1* locus significantly reduces nematode multiplication, its deployment is complicated by linkage to the high-yellow pigment allele *Psy-A1t*, which is undesirable in bread-making wheat (Jayatilake et al. 2013). Additional quantitative trait loci (QTL) have been mapped on wheat chromosomes 1A, 2A, 2B, 4D, 5A, 6B, and 7D, but explained < 15% of the phenotypic variation (Mulki et al. 2013; Dababat et al. 2016; Thompson et al. 2017). Despite progress in QTL discovery, breeding uptake in wheat has been slow because: (i) extraction and microscopic counting of nematodes from soil and root samples based on morphological characteristics is laborious (Smiley 2021), and (ii) wheat has a complex polyploid genome and a scarcity of favorable alleles in elite gene pools, necessitating introgression from closely related or wild germplasm (Wen et al. 2018). Consequently, there is a critical need for the development of molecular markers linked to nematode resistance to facilitate marker-assisted selection and accelerate breeding efforts (Singh et al. 2023).

Triticale (× *Triticosecale* Wittm., 2*n* = 6*x* = 42, AABBRR genomes), a synthetic hybrid generated by combining the genomes of wheat (*Triticum* spp., AABB genomes) and rye (*Secale cereale* L., RR genomes), represents a promising yet underexploited reservoir of nematode resistance for cereal improvement (Ayalew et al. 2018; Mergoum et al. 2019). In particular, rye is known for its broad resistance to various biotic and abiotic stresses, which triticale inherits to a large extent (Zeller and Hsam 1983; Mergoum et al. 2004; Saulescu et al. 2011). Breeders have long utilized triticale as a bridge to introgress valuable rye-derived traits, including resistance to pests and pathogens, into wheat germplasm (Friebe et al. 1996; Wang et al. 2023). Although global acreage of triticale remains modest (FAOSTAT 2024), interest is growing due to its versatility in forage, silage, and cover cropping systems, alongside coordinated breeding efforts focused on disease resistance and grain quality (Ayalew et al. 2018; Mergoum et al. 2019). Notably, several studies have also demonstrated the potential of triticale as a source of resistance to RLNs (Farsi et al. 1995; Vanstone et al. 1998; Taylor et al. 2001). Farsi et al. (1995) reported that triticale cultivars (Abacus and Muir) had significantly fewer nematodes per gram of root and per plant compared to susceptible wheat cultivars. In Australian field trials, Vanstone et al. (1998) observed that triticale varieties harbored lower populations of *P. neglectus* than wheat, barley, and oats, indicating inherent resistance mechanisms. Taylor et al. (2001) confirmed that all triticale cultivars screened were resistant to *P. neglectus*, whereas resistance in wheat was limited. Our recent greenhouse experiments have further validated the resistance potential of triticale, with the cultivar Villax St. Jose consistently demonstrating significantly lower nematode reproduction than a panel of elite wheat cultivars and germplasm lines (Singh et al. 2023). Building on this observation, the present study utilizes the Siskiyou × Villax St. Jose (Wen et al. 2018) recombinant inbred line (RIL) population to (i) identify and map QTL associated with reduced nematode reproduction and (ii) convert tightly linked single-nucleotide polymorphisms (SNPs) into Kompetitive allele-specific PCR (KASP) assays. These assays constitute a rapid genotyping resource that can be further validated and integrated into triticale and wheat breeding pipelines aimed at enhancing RLN resistance. By filling a critical gap in our understanding of RLN resistance genetics in an under-utilized cereal, this work aims to broaden the genetic foundation for sustainable nematode management in cereal production.

## Materials and methods

### Nematode population collection, processing, and species confirmation

Soil samples were collected from North Dakota wheat fields known to be infested with *P. neglectus* following a protocol previously described by Singh et al. (2023). In brief, sampling occurred during the cropping season or immediately after harvest of the crop. Using a standard soil probe (2.5 cm diameter and 30 cm depth), samples were taken in a zig-zag pattern across the fields, with approximately 25–30 soil cores per field. Samples were combined into a composite and transported to the Nematology Laboratory at North Dakota State University (NDSU), Fargo, ND. Ten subsamples of 200 grams (g) each were taken from the well-mixed composite soil for nematode extraction using the Whitehead tray method (Whitehead and Hemming 1965) to determine initial population densities. This technique relies on the active migration of nematodes from moist soil into water. Each subsample was filtered through a 250 µm aperture sieve, and nematodes were collected on a 20 µm sieve, which was then concentrated to 20–25 mL in a 50-mL vial (Capitol Vial Inc., Auburn, AL, USA). Nematodes were observed and identified to the genus level based on morphological features using a compound microscope (Zeiss Axiovert 25, Carl Zeiss Microscopy, NY, USA) and a Peters 1-mL gridded slide (Chalex Corporation, Portland, OR, USA). Molecular identification was performed by using *P. neglectus*-specific PCR primers as described in Yan et al. (2008).

### Rearing pure populations of *P. neglectus*

Pure cultures of *P. neglectus* were generated and maintained in monoxenic carrot disk cultures following Lawn and Noel (1986) with minor modifications. Organically grown and fresh, unblemished carrots were washed, rinsed, and surface-sterilized in 10% bleach for 30 min in a laminar-flow hood. Using sterile instruments, carrots were peeled and sliced into 6–10 mm disks, placed in sterile Petri dishes, sealed, and incubated at 22 °C in the dark. Disks were preconditioned for 1–2 weeks until calli formed. Individual nematodes were surface-sterilized in 0.01% streptomycin at 4 °C overnight, selected under a dissecting microscope, and transferred to disks. The disks were placed in sterile Petri dishes (Thermo Fisher Scientific, Waltham, MA, USA), sealed with Parafilm, and incubated in the dark at 22 °C. The carrot cultures were incubated in the dark at 22 °C for up to six months, with weekly evaluations of carrot disk stability and nematode reproduction. Once the carrot disks reached six months or before decayed, nematodes were harvested. The disks were thinly sliced and soaked in distilled water in a Petri dish. After 3–4 h, the water containing the nematodes was filtered through a 20 µm sieve, and the nematodes were collected in a 50-mL vial. The nematode suspension was refrigerated (~ 4 °C) until use. To increase inoculum, pre-germinated seeds of the spring wheat cultivar Alpowa (susceptible to *P. neglectus*; Smiley et al. 2014; Singh et al. 2023) were used. Plants were grown in plastic pots containing 1 kg of pasteurized sandy-loam soil (67% sand, 18% silt, 15% clay). The soil was prepared in-house by blending river sand with field soil, and its particle-size distribution was verified by a commercial laboratory (Agvise Laboratories, Northwood, ND, USA). One week after planting, nematodes harvested from carrot cultures were inoculated near the roots by pipetting the suspension into four small holes around each plant; holes were backfilled with moist soil. Pots were watered lightly immediately after inoculation to settle the inoculum and then maintained at moderate moisture for 48–72 h to support nematode movement while avoiding leaching. Thereafter, pots were top watered (with low pressure) as needed to keep soil moisture consistent. Plants were grown for 14 weeks in the Jack Dalrymple Agricultural Research Complex, NDSU, Fargo, ND, USA at 22 °C under a 16-h photoperiod.

### Plant materials

We used a triticale mapping population segregating for *P. neglectus* to map resistance loci (Singh et al. 2023). The population was derived from a cross between the susceptible triticale cultivar Siskiyou (L12G09) and the moderately resistant triticale accession Villax St. Jose (L12G18). Both parents are hexaploid (2*n* = 6*x* = 42, AABBRR) spring types. Siskiyou, developed collaboratively by the International Maize and Wheat Improvement Center in Mexico (CIMMYT) and the University of California, Davis, was released as a cultivar in California (Qualset et al. 1985). Villax St. Jose (PI 428848) is a Moroccan cultivar (Kuleung et al. 2006). The development of this mapping population was previously detailed by Wen et al. (2018). In this study, 137 F_6_ RILs were screened for resistance to *P. neglectus*. The *P. neglectus*-susceptible wheat cultivar Alpowa, the resistant Iranian wheat landrace AUS28451, and unplanted inoculated and non-inoculated controls were used as control standards (Smiley and Machado 2009; Singh et al. 2023).

### Experimental design and assessment for *P. neglectus* resistance

The phenotypic evaluations of the RIL population for resistance to *P. neglectus* were performed in two independent greenhouse experiments in 2020 and 2024 at the Jack Dalrymple Agricultural Research Complex, NDSU, Fargo, ND, USA. A single pre-germinated seed of each line was planted in a cone container filled with 150 g of pasteurized sandy loam soil (67% sand, 18% silt, and 15% clay; as described above) with approximately 2 g of ‘Osmocote Plus’ 15-19-12 fertilizer (Scotts Sierra Horticultural Product Company, Maysville, OH, USA). Within each experiment, five biological replicates were grown per RIL and control. Each cone container served as one experimental replicate. In total, we evaluated 141 lines per experiment (137 F_6_ RILs, two parents [Siskiyou and Villax St. Jose], and two wheat checks [Alpowa and AUS28451]). In addition, two unplanted controls (inoculated and non-inoculated) were included, each with five replicates. The cone containers were placed in RL98 trays (Stuewe & Sons, Inc., Corvallis, OR, USA) and arranged in a completely randomized design. This design yielded 715 planted experimental units per experiment and 1430 units across both experiments. Plants were maintained in the greenhouse for 14 weeks at an average temperature of 22 °C with a 16-h photoperiod.

In both experiments, inoculum consisted of mixed life stages of *P. neglectus* (eggs, second-stage juveniles, and adults) pooled from two sources: monoxenic carrot-disk cultures and greenhouse-propagated populations maintained on susceptible wheat (cv. Alpowa) in pasteurized soil (described above). Seven days after planting, two 2 cm wells were made beside each seedling and inoculated with water suspensions of 300 ± 10 *P. neglectus* (eggs, J2, and adults) per cone for each experiment. Cone containers were top watered (with low pressure) as needed to keep soil moisture consistent for the first 7 days and then resumed carefully to avoid leaching of the nematodes. After 14 weeks, the entire root systems and the surrounding soil were harvested, stored at 4 °C, thoroughly mixed, and processed on trays using the Whitehead tray extraction method (Whitehead and Hemming 1965; Singh et al. 2023). After 48 h, nematodes were collected and counted under a compound microscope (Zeiss Axiovert 25, Carl Zeiss Microscopy, NY, USA) to determine the final (postharvest) population densities (Pf) of *P. neglectus*.

Following the standardized phenotyping strategy proposed by Singh et al. (2023), Pf values were converted to relative values by normalizing against the susceptible check Alpowa within each experiment. When single experiments were analyzed, the numerator and denominator were the replicate means of Pf; in the combined analysis, they were the best linear unbiased predictors (BLUPs) described below. This standardization helps account for experiment-specific variability and allows consistent comparison across genotypes and runs. Relative Pf (%) was calculated as:

$$\text{Relative Pf or BLUPs}=\frac{\text{Pf }(\text{or BLUPs})\text{ from tested genotype}\times 100}{\text{Pf }(\text{or BLUPs})\text{ from susceptible check Alpowa}}$$

### Phenotypic data analysis

To determine whether data from the two greenhouse experiments could be analyzed together, the homogeneity of error variances with Levene’s test (PROC GLM, SAS 9.4, SAS Institute, Cary, NC, USA) was examined. First, variance among the five replicates of every RIL was examined within each experiment; no significant heterogeneity was detected (*p* > 0.05). Second, genotype means were compared between experiments, and again Levene’s test revealed no variance differences (*p* > 0.05). Because both within- and between-experiment variances were homogeneous, replicate values were averaged to give one phenotype per RIL per experiment. Genotype means from Experiments 1 and 2 were then correlated with Pearson’s *r* using ‘*cor.test*’ in R Studio v4.3.2 (R Core Team 2024) to quantify stability of the trait across experiments. A two-way analysis of variance (ANOVA) was performed to quantify the effects of experiment, genotype and their interaction:

$${{Y}_{ijk}= \mu +{E}_{i} +{G}_{j} +{(G\times E)}_{ij} +\varepsilon }_{ijk}$$

where *Y*_*ijk*_ is the relative Pf of the *k*th replicate of genotype *j* in experiment *i*; *μ* is the overall mean; *E*_*i*_ is the fixed effect of the *i*th experiment; *G*_*j*_ is the fixed effect of genotype *j*; (*G* × *E*)_*ij*_ is the fixed interaction term; and *ε*_*ijk*_ is the residual error. ANOVA revealed highly significant effects (*p* < 0.0001) of experiment, genotype, and their interaction, indicating that genotypes responded differentially across years. Although the experiment effect influenced trait means, replicate variances remained homogeneous, validating subsequent mixed-model adjustment. To account for experimental effect and genotype × experiment (*G* × *E*) interaction, we calculated BLUPs for each RIL or genotype using a linear mixed model fitted in the *lme4* package in R Studio v4.3.2:

$${{Y}_{ijkl}= \mu +{E}_{i} +{G}_{j} +{{(E\times R)}_{ik}+ (G\times E)}_{ij} + \varepsilon }_{ijkl}$$

where *E*_*i*_ is a fixed effect (experiment), (*E* × *R*)_*ik*_ is replication nested within experiment, *G*_*j*_ is a random genotypic effect, and (*G* × *E*)_*ij*_ is a random genotype by experiment interaction. Parents and check cultivars were retained during model fitting to stabilize variance estimates but were excluded for downstream analysis. The resulting BLUPs provide experiment-adjusted, shrinkage-corrected estimates of genotypic performance and were used as phenotypic input in QTL analysis. Variance components from the mixed model were also used to compute broad-sense heritability (*H*^2^) on a line-mean basis,

$${H}^{2} = \frac{{\sigma }_{G}^{2}}{{\sigma }_{G}^{2}+ \frac{{\sigma }_{GE}^{2}}{e}+ \frac{{\sigma }_{\varepsilon }^{2}}{re}}$$

where ${\sigma }_{G}^{2}$ represents a genotypic variance, ${\sigma }_{GE}^{2}$ is a genotype × experiment interaction variance, ${\sigma }_{\varepsilon }^{2}$ is the residual variance, *r* is the number of replicates per experiment, and *e* represents the number of experiments.

### Genotyping-by-sequencing, SNP filtering, and linkage map construction

Raw genotyping-by-sequencing (GBS) reads for RILs derived from the Siskiyou × Villax St. Jose cross, together with both parents, were retrieved from the NCBI Sequence Read Archive (SRA Accession: SRR5821028). The data were then compared to two reference genomes. The first genome was an artificial triticale genome generated by combining the ‘A’ and ‘B’ genomes of the wheat landrace Chinese Spring (IWGSC CS v1.0) and the ‘R’ genome of the rye line Lo7 version 2 (Bauer et al. 2017). The first artificial triticale genome provides valuable context relative to established, well-curated, and gene-annotated reference genomes of wheat and rye. The second genome used a newly assembled genome of the triticale cultivar Siskiyou (unpublished, Dr. Zhaohui Liu). The second genome provides a more accurate analysis of genomic information in the context of a true triticale cultivar and one of the parental lines of the mapping population.

The data for the artificial triticale genome were analyzed with the reference-free TASSEL UNEAK pipeline (Wen et al. 2018). The genome of Siskiyou was analyzed using the TASSEL 5.0 GBSv2 pipeline (Glaubitz et al. 2014). Barcode demultiplexing, tag generation, alignment to the reference genome using Bowtie2 (version 2.4.5), SNP discovery, and variant calling were performed within the TASSEL (version 5.2.96) GBSv2 command-line interface (Glaubitz et al. 2014). The resulting variant call file (VCF) was filtered using VCFtools (version 0.1.16) and TASSEL (version 5.2.96; Bradbury et al. 2007) software. SNPs were retained if they met the following criteria: (i) minor allele frequency (MAF) ≥ 0.35, (ii) missing data ≤ 50%, and (iii) heterozygosity ≤ 10%. Individuals with > 70% missing data were excluded, and any residual heterozygous genotype calls were converted to missing calls (Singh 2025).

Co-segregating and redundant loci were removed to retain unique, high-confidence SNPs for linkage analysis. Linkage maps were constructed using MapDisto v2.1.7 (Heffelfinger et al. 2017), following the procedures described by Wen et al. (2018). Marker grouping was performed using the < Find Linkage Groups > command with a logarithm of odds (LOD) threshold of 3.0 and a maximum recombination frequency (*R*_max_) of 30.0. The Kosambi mapping function (Kosambi 1944) was used to convert recombination frequencies into genetic distances. Within each linkage group, marker ordering and map refinement were done using < Order a linkagegroup > , < Check inversions > , < Ripple order > , and < Drop locus > commands as described by Wen et al. (2018) and Acharya et al. (2024).

### QTL analysis and KASP marker development

QTL mapping was performed in QGene v4.3.10 (Joehanes and Nelson 2008) using a scanning interval of 10 (equivalent to a 1.0 cM step size) to enable high-resolution detection of QTL across the linkage map (Singh 2025). The single-trait multiple interval mapping (STMIM) function was used to identify QTLs significantly associated with *P. neglectus* resistance. Phenotypic inputs were relative Pf (%) for the single-experiment analyses and relative BLUPs (%) for the combined analysis. A permutation test consisting of 1000 iterations was used to determine a LOD threshold for STMIM at a significance level of 0.05. The coefficient of determination (*R*^2^) was used to estimate the phenotypic variation that the identified QTL explained. The initial genome-wide scan identified a significant QTL on chromosome 5R.

SNP‐containing GBS tags flanking the 5R QTL peak were converted into KASP assays with PolyMarker (Ramirez-Gonzalez et al. 2015). PolyMarker facilitated the alignment of GBS tag sequences to Lo7 v2 genomic assembly, resulting in the generation of two allele-specific primers and one common primer for each assay. Primer candidates were screened in silico against both the Lo7 and Weining rye genomes (Rabanus-Wallace et al. 2021; Li et al. 2021) to confirm their single-copy specificity. For tags that failed PolyMarker’s design criteria, primers were manually redesigned using Primer3 (Seneviratne et al. 2024; Running et al. 2025), again anchored to orthologous regions in the Lo7 and Weining genomic assemblies. All primer pairs were remapped to the triticale genome of Siskiyou to confirm their unique placement and correct orientation. The results showed complete concordance with the rye-based designs.

The resulting KASP assays were evaluated in the parental lines and the RIL population and then incorporated into the chromosome 5R linkage map. The QTL analysis was finally repeated using all markers, including the KASP markers, to improve the accuracy of the position and effect estimates of the 5R locus. To compare nematode reproduction between allelic classes at diagnostic markers, Welch’s *t* tests were performed on relative Pf or BLUP values using the function ‘*scipy.stats.ttest_ind*’ (SciPy v1.11.4 in Python), with unequal variances assumed (*equal_var* = *False*) for each experiment and for the combined dataset.

## Results

### Reactions of triticale parents and RILs to *P. neglectus*

**Fig. 1:**
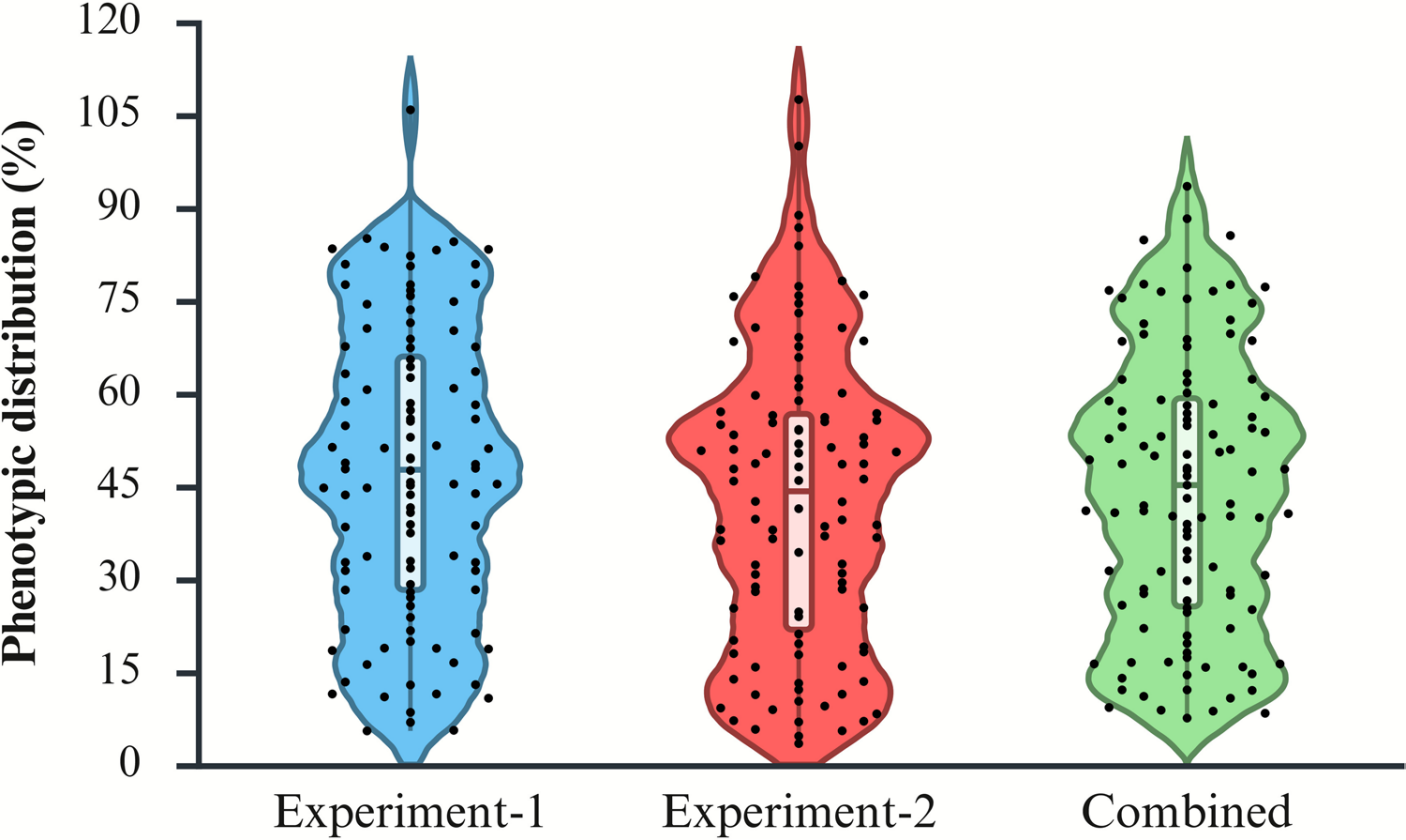
Phenotypic distribution of recombinant inbred lines (RILs) from the Siskiyou × Villax St. Jose triticale population for resistance to *Pratylenchus neglectus*. Violin plots show the frequency and spread of relative nematode population densities (%) for Experiment 1 (blue), Experiment 2 (rose), and the combined dataset (light green). Each black dot represents an individual RIL, and the white box inside each violin indicates the interquartile range with the median line. Data for Experiments 1 and 2 represent relative Pf (%) from independent greenhouse assays, while the combined dataset represents relative BLUPs (%) estimated across both experiments. Lower values indicate greater *P. neglectus* resistance relative to the susceptible check Alpowa

The phenotypic assay confirmed the expected reaction classes of the reference genotypes: AUS28451 exhibited a highly resistant response (relative Pf = 8.8%), Alpowa was susceptible (100.0%), Villax St. Jose was moderately resistant (29.7%), and Siskiyou was susceptible (80.3%) (Table 1). Among the 137 RILs, relative Pf ranged from 2.7 to 113.4% in Experiment 1 and 2.9 to 109.8% in Experiment 2, with population means of 47.0% and 42.7%, respectively (Supplementary Table S1). Environment-adjusted BLUPs, integrating both experiments, ranged from 7.5 to 93.4% with a mean of 46%. The distribution of relative Pf or BLUP values approximated a normal distribution, with transgressive segregants observed on both tails of the curve (Fig. 1). Genotype performance was strongly conserved across years (*r* = 0.796, *p* < 0.001), and the mixed-model analysis yielded a broad-sense heritability (*H*^2^) of 0.903.

**Table 1:** The response of parental lines and RIL to *Pratylenchus neglectus* in the Siskiyou × Villax St. Jose triticale population

Category	Descriptor^x^	Exp-1 (%)^y^	Exp-2 (%)^y^	Combined (%)^z^
Reaction of parental lines	Siskiyou (Susceptible)	85.00	75.62	80.27
	Villax St. Jose (Resistant)	29.14	30.74	29.72
Reaction of recombinant inbred lines (RILs)	Minimum value	5.46	3.42	7.53
	Maximum value	105.79	107.45	93.43
	Mean value	46.98	42.37	43.93

^x^RIL statistics summarize RILs; each entry (parents, checks, and RILs) had five biological replicates per experiment

^y^Exp = Experiment. Experiment-1 and Experiment-2 were independent greenhouse assays conducted at the Jack Dalrymple Agricultural Research Complex, North Dakota State University (Fargo, ND, USA) in 2020 and 2024, respectively. Exp-1 (%) and Exp-2 (%) are Relative Pf (%), calculated within each experiment as: 100 × [Pf of the genotype] / [Pf of Alpowa]. Pf is the postharvest *P. neglectus* population densities

^z^Combined (%) are relative BLUPs from a linear mixed model across both experiments: Relative BLUP (%) = 100 × [genotype BLUP] / [Alpowa BLUP]. The model used genotype as a random effect, experiment as a fixed effect, and replicate nested within experiment as a random term

### Linkage map construction

Re-analysis of the raw fastq files originally reported by Wen et al. (2018) generated 120.5 million reads, which, after alignment to the Siskiyou reference genome, yielded 1.04 million unique 64-bp tags. SNP discovery identified 118,903 biallelic sites. Sequential filtering for genotype call-rate (≥ 50%), minor-allele frequency (≥ 0.35), and residual heterozygosity (≤ 10%) (5750 polymorphic SNPs; Supplementary Table S2), followed by the removal of redundant or co-segregating loci, yielded 1054 non-redundant, high-confidence SNPs (Supplementary Table S3). These SNPs, together with seven chromosome-5R SSRs, provided 1061 markers for linkage analysis. The marker set resolved into the expected 21 linkage groups, comprising 14 wheat (AA and BB genomes) and 7 rye (RR genome), which spanned 2745.9 cM (Supplementary Figure S1). The composite map contained 1111.7 cM in the AA genome, 915.4 cM in the BB genome, and 718.9 cM in the RR genome, resulting in a genome-wide mean spacing of 2.58 cM per marker (Supplementary Table S4). Individual linkage groups ranged from 80.6 cM (2R) to 213.2 cM (7A). Marker density varied from 2.13 cM/marker (5R) to 3.80 cM/marker (5A).

### QTL identification on chromosome 5R

**Fig. 2:**
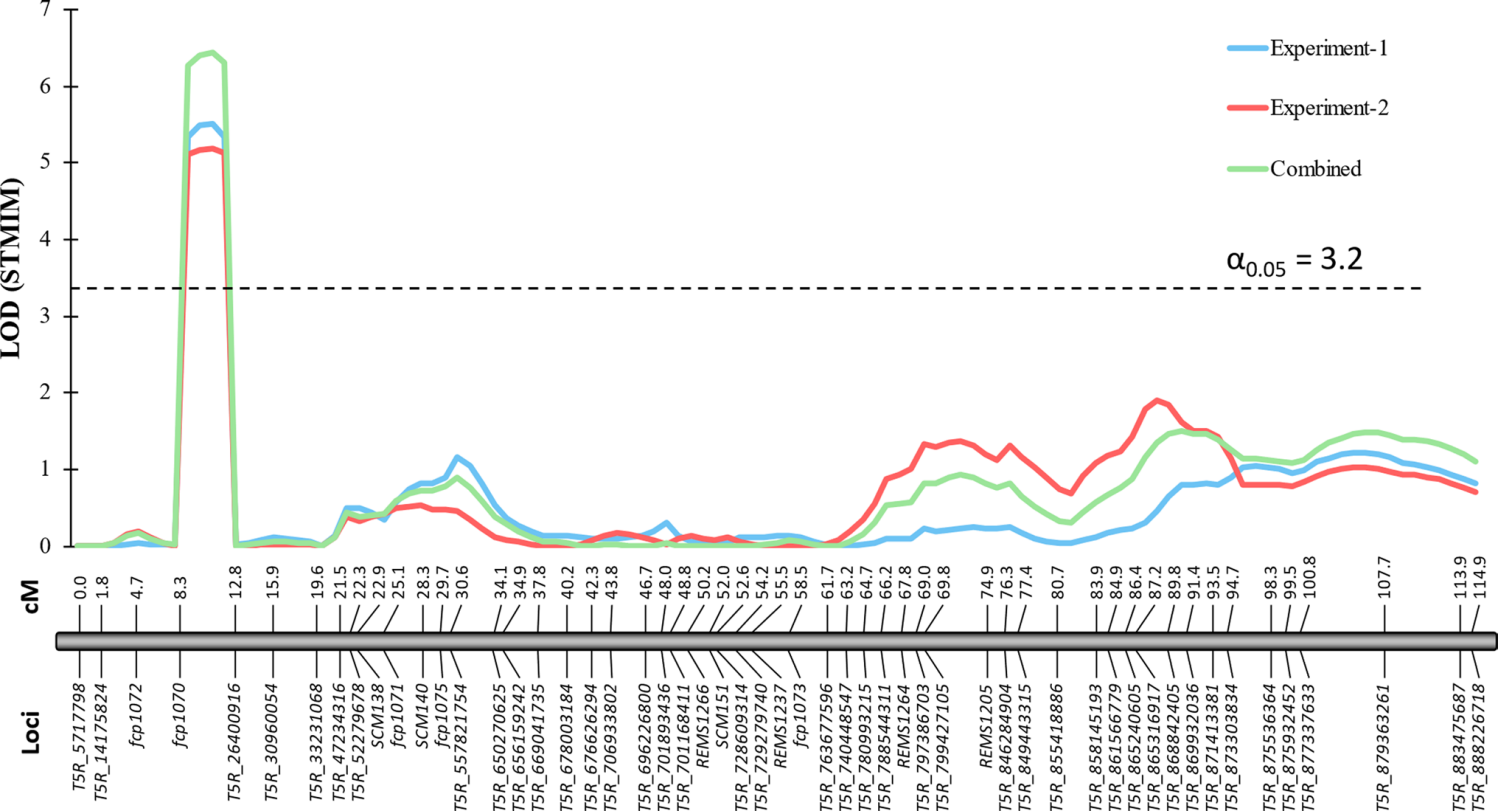
Single-trait multiple-interval mapping (STMIM) of a quantitative trait locus (QTL) associated with resistance to *Pratylenchus neglectus* on chromosome 5R in the Siskiyou × Villax St. Jose RIL population. LOD profiles are presented for Experiment-1 (blue), Experiment-2 (rose), and the combined dataset (light green). Marker names are shown below the chromosome axis, with corresponding genetic distances (in cM) indicated above. The horizontal dashed line represents the significance threshold (LOD = 3.2) at a genome-wide *α* = 0.05

A genome‐wide scan with STMIM detected a single locus exceeding the 5% experiment-wise permutation threshold (LOD = 3.2) (Supplementary Figure S2). The peak occurred at 11 cM with a LOD of 6.5 and a generalized *R*^*2*^ of 0.196 in the combined analysis (Fig. 2). Adjacent positions 9–12 cM also surpassed the threshold (LOD ≥ 6.3), delimiting a 4 cM interval that accounted for ~ 20% of the phenotypic variance in BLUPs and ~ 16–17% in the individual experiment means. No additional significant loci were detected elsewhere in the genome, indicating that a single, moderate-effect QTL largely conditions resistance in this population on 5R, hereafter designated as *QRlnn.ndsu-5R*.

### KASP marker associated with *QRlnn.ndsu-5R*

**Fig. 3:**
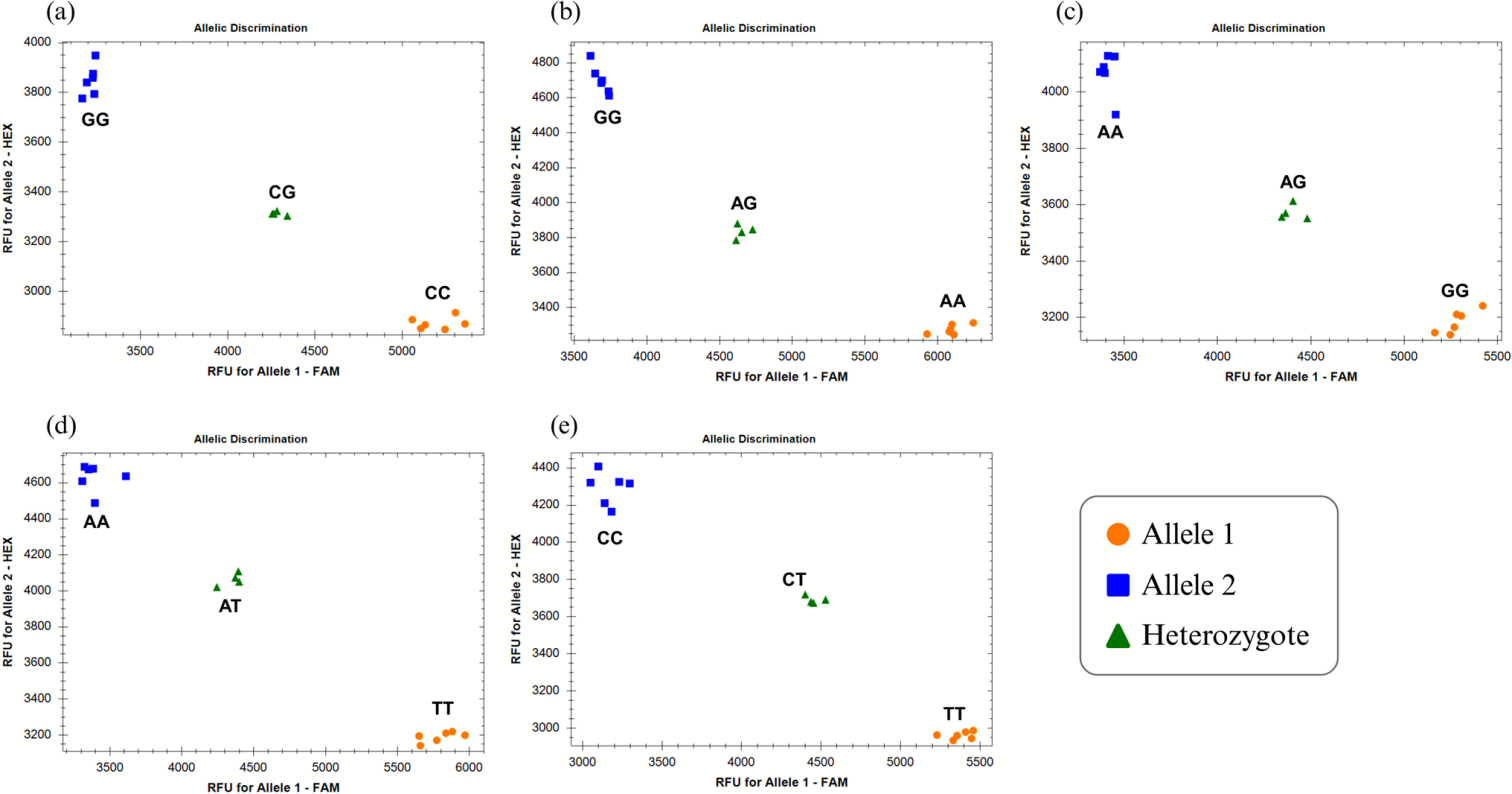
Allelic discrimination plots of the five KASP markers mapped on chromosome 5R in the Siskiyou × Villax St. Jose population (**a** *fcp1070*, **b** *fcp1071*, **c** *fcp1072*, **d** *fcp1073*, and **e** *fcp1075*). Each panel displays relative fluorescence units (RFU) for Allele 1 (FAM) on the x-axis and Allele 2 (HEX) on the y-axis. The orange dots and blue squares represent homozygous allele 1 and homozygous allele 2, respectively, and the green triangles represent heterozygote alleles

**Fig. 4:**
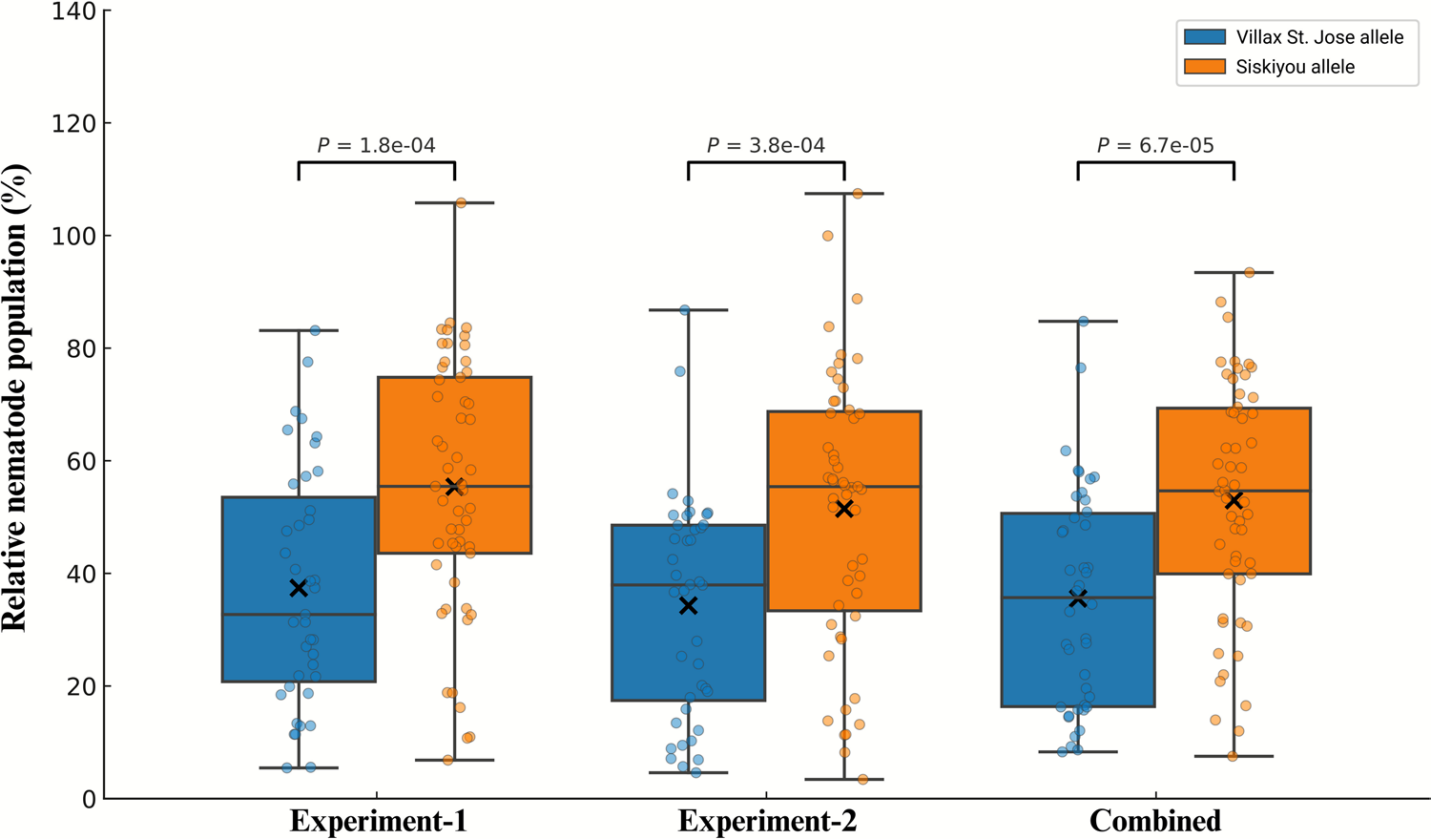
Boxplots showing the effect of *fcp1070* alleles on *Pratylenchus neglectus* resistance in the Siskiyou × Villax St. Jose recombinant inbred line (RIL) population. RILs were grouped by genotype at marker *fcp1070* and evaluated for relative nematode population (%), where values represent the final nematode population density (Pf) relative to the susceptible parent for Experiment-1 and Experiment-2, and relative best linear unbiased predictors (BLUPs) for the combined dataset. Blue boxes indicate individuals carrying the Villax St. Jose allele, and orange boxes indicate those with the Siskiyou allele. Horizontal lines within boxes represent medians, black crosses denote means, and individual dots represent RIL values. *p* values are from Welch’s *t* tests, indicating significant differences in nematode resistance between genotypic groups (*p* < 0.0001) across all datasets

Of the five chromosome-5R SNPs converted to KASP assays (Table 2), *fcp1070* (derived from GBS tag *TP4673*) is the most informative for *P. neglectus* resistance. This marker lies only ~ 3 cM from the maximum‐LOD position of *QRlnn.ndsu-5R* and falls well inside the 4 cM interval. Genotyping all 137 RILs and both parents produced unambiguous biallelic clusters (Fig. 3). Lines that possessed the Villax St. Jose allele exhibited a mean relative BLUP of 35.5%, whereas lines with the Siskiyou allele averaged 53.0%. The difference in nematode reproduction was highly significant (Welch’s *t*-statistic = −4.18, *p* = 6.7 × 10⁻^5^) (Fig. 4). A similar separation between genotype classes was also observed when the two greenhouse experiments were analyzed separately, confirming the stability of the marker effect across environments.

**Table 2:** Kompetitive allele-specific PCR (KASP) markers mapped to chromosome 5R in the Siskiyou × Villax St. Jose triticale recombinant inbred line population

KASP marker^w^	SNP^x^	Primer names	Primer sequences (5′→3′)^y^	Physical position (Mbp)^z^
*fcp1072*		*fcp1072*-FAM	gaaggtgaccaagttcatgctTGCTGCAGATTCATGAAGTGG	
	G/A	*fcp1072*-HEX	gaaggtcggagtcaacggattTGCTGCAGATTCATGAAGTGA	*20,200,087*
		*fcp1072*-Com	GTTGGTTCTCTGTCCTCTGTATCC	
*fcp1070*		*fcp1070*-FAM	gaaggtgaccaagttcatgctGATTGGGTGCGTGTGACATC	
	G/C	*fcp1070*-HEX	gaaggtcggagtcaacggattGATTGGGTGCGTGTGACATG	*26,708,526*
		*fcp1070*-Com	CCCACCATGTGCCAAAATAATTC	
*fcp1071*		*fcp1071*-FAM	gaaggtgaccaagttcatgctGCCTGGACGCCTATTTATCCA	
	G/A	*fcp1071*-HEX	gaaggtcggagtcaacggattGCCTGGACGCCTATTTATCCG	*365,423,142*
		*fcp1071*-Com	GGTTTCTTTGGTGCTGCAGAT	
*fcp1075*		*fcp1075*-FAM	gaaggtgaccaagttcatgctAAGGTTGTCTGCAGCTCTCT	
	C/T	*fcp1075*-HEX	gaaggtcggagtcaacggattAAGGTTGTCTGCAGCTCTCC	*561,038,158*
		*fcp1075*-Com	CCTATGGGAGTCTTGGCGAC	
*fcp1073*		*fcp1073*-FAM	gaaggtgaccaagttcatgctGTCAACGTCTCTGCAGCCT	
	T/A	*fcp1073*-HEX	gaaggtcggagtcaacggattGTCAACGTCTCTGCAGCCA	*739,373,203*
		*fcp1073*-Com	AGCTCTGCTGAAACCCGAATAT	

^w^KASP = Kompetitive allele-specific PCR, a fluorescence-based genotyping method using two allele-specific forward primers (labeled with FAM or HEX tails) and one common reverse primer

^x^SNP = single-nucleotide polymorphism. The variant base is shown for each marker. Siskiyou allele/Villax St. Jose allele

^y^Primer sequences are shown in the 5′→3′ direction. Lowercase letters at the beginning of FAM- and HEX-labeled forward primers represent the standard tail sequences recommended by the KASP chemistry for fluorophore binding; uppercase letters represent the locus-specific portion of the primer sequence

^z^Physical positions (in megabase pairs, Mbp) are based on the Siskiyou triticale reference genome assembly

## Discussion

RLNs continue to impose substantial yield penalties in rainfed cereals because nematicides are expensive and environmentally concerning, and crop rotation is unreliable since *P. neglectus* can survive on a wide range of hosts (Smiley 2021). Therefore, genetic resistance remains the most durable defense strategy (Singh et al. 2023). After decades of screening, wheat breeding still relies on a single major locus (*Rlnn1*) along with a few small-effect QTLs, which together leave significant nematode populations in the soil (Williams et al. 2002; Zwart et al. 2010; Mulki et al. 2013; Dababat et al. 2016). Rye (*S. cereale*) is well known for broad disease resistance, and its amphiploid derivative, triticale (AABBRR genome), offers a practical bridge for deploying rye alleles into wheat (Wen et al. 2018). To our knowledge, no RLN resistance locus has been genetically mapped in rye or triticale. In this study, we identified *QRlnn.ndsu-5R* on chromosome 5R, which helped address this gap and provided genetic evidence from a rye-derived resistance source for cereal improvement.

Although *QRlnn.ndsu-5R* explained about 20% of the phenotypic variance, the near-normal distribution of the phenotypic data, together with transgressive RILs that exceeded both parents, indicated that additional loci likely contributed to resistance. Such transgression is consistent with complementary small-effect alleles from both parents. We used the existing Siskiyou × Villax St. Jose population, which contains 137 F_6_ RILs as previously described (Wen et al. 2018). RLN phenotyping is very labor- and space-intensive in that each line required five biological replicates and a 14-week greenhouse cycle in containment conditions, which constrained the feasibility of increasing the number of lines in this study. This design provided stable Pf estimates but offered limited power to detect very small-effect loci, so some modifiers may have remained below the detection threshold (Supplementary Figure S2). The consistent detection of *QRlnn.ndsu-5R* across both independent experiments supports the reliability and stability of this major locus. In future, expanding population size, increasing marker density around 5R, and evaluating across additional environments will help resolve minor-effect loci and test for possible epistasis with *QRlnn.ndsu-5R*.

Screening for nematode resistance is laborious, time-consuming, and expensive, which is the major constraint in nematode resistance breeding (Smiley 2021). Depending upon the availability of resources, a single screening experiment may require 5–6 months to generate phenotypic data (Singh et al. 2023; Thompson et al. 2017). Thus, genotypic selection using molecular markers can be a valuable alternative to the lengthy and labor-intensive process of resistance phenotyping (Singh et al. 2023). The KASP markers we have developed can serve as valuable tools for monitoring the introgression of resistance QTL in *P. neglectus.* Genotyping with *fcp1070* partitioned the 137 RILs into two allelic classes, whose mean nematode multiplications differed by ~ 33% (*p* < 0.001), closely matching the variance attributed to *QRlnn.ndsu-5R*. Genotypic analysis, in conjunction with phenotypic screenings for resistance to *P. neglectus* in greenhouse environments, is expected to facilitate the development of germplasm exhibiting nematode resistance. In addition, as chromosome-scale triticale reference (Siskiyou; unpublished) resources become available, additional SNPs within the QTL region can be developed into KASP assays. Development of flanking-marker pairs spanning the QTL region could reduce recombination-mediated loss of the locus in marker-assisted selection. Increasing marker density to saturate this region will improve genetic and physical map resolution and lay the foundation for fine-mapping the resistance determinant (Singh 2025). Further fine-mapping and cloning of this QTL can help develop robust diagnostic markers for marker-assisted selection in breeding programs and deepen our understanding of the genetic mechanisms underlying nematode resistance.

In wheat, the major locus *Rlnn1* on chromosome 7AL is located within an interval that includes multiple disease resistance loci [e.g., *Pm1a* (powdery mildew), *Lr20* (leaf rust), and *Sr15* (stem rust)] and clusters of nucleotide-binding leucine-rich repeat (NLR)-type receptors (Jayatilake et al. 2013). Additional wheat *P. thornei* QTLs on chromosomes 6D and 2B have also been fine-mapped, with intervals indicating NLR or kinase receptor candidates expressed in roots (Rahman et al. 2020). In barley, the principal *P. thornei* QTL on 7H includes two defense-related receptor-like protein kinases (RLKs) with higher basal expression in resistant lines (Mather et al. 2024). In this study, we also examined the physical interval defining *QRlnn.ndsu-5R*, which spans approximately 10 Mbp (from 20.20 Mbp to 30.96 Mbp) on the triticale (Siskiyou) reference genome. This region contains 140 high-confidence genes (unpublished, Dr. Zhaohui Liu), including three NLR-type resistance-gene analogs and two coiled-coil RLKs. Based on existing literature, we hypothesize that immune-receptor genes could be potential candidates within this region. However, no RLN resistance gene has yet been cloned, and other mechanisms (e.g., genes involved in cell wall remodeling, detoxification/redox pathways, transport, or hormone signaling) cannot be ruled out. Further delineation by fine mapping is needed to narrow down the candidate gene region and resolve the genetic basis of RLN resistance in this interval.

Rye chromatin has repeatedly lifted wheat disease resistance to new plateaus; for example, the 1RS.1BL/1AL translocations carrying *Lr26* (leaf rust), *Sr31* (stem rust), *Pm8* (powdery mildew), and *Yr9* (yellow rust) are now present in > 1000 cultivars worldwide (Schlegel 2023; Wang et al. 2023). Moreover, the rye gene *CreR* on chromosome 6R has been introgressed to combat cereal cyst nematodes (Dundas et al. 2001). Notably, for 5R specifically, a compensating T5AS.5RL Robertsonian translocation has been produced, demonstrating that 5R chromatin can be stabilized in wheat; at the same time, such arm-level transfers exemplify the potential for extensive alien segments and linkage drag (Efremova et al. 2014; Chumanova et al. 2014). Meanwhile, a 5R segment harboring *Xct1* confers dominant resistance to bacterial leaf streak in triticale and could also be transferred into bread wheat via *ph1b*-mediated homoeologous recombination (Wen et al. 2018). At the level of homoeology, 5R is broadly related to wheat group-5 chromosomes (5A/5B/5D), and crossovers in Triticeae are strongly biased toward the distal ends, characteristics that facilitate recovery of small recombinant segments when the target interval is away from the pericentromeres (Efremova et al. 2014; Chumanova et al. 2014; Li et al. 2016). *QRlnn.ndsu-5R*, therefore, joins a growing list of rye-derived factors that tackle otherwise recalcitrant threats in wheat. Because *QRlnn.ndsu-5R* lies within a modest ~ 10-Mb interval, there is a realistic prospect of moving only a minimal rye fragment, mitigating the linkage drag that has occasionally accompanied larger translocations such as 1RS (Wang et al. 2023). Marker-assisted backcrossing with *fcp1070*, followed by targeted recombination in *ph1b* or CRISPR-enabled chromosome-engineering backgrounds, should enable precise introgression and subsequent trimming of unwanted rye DNA. To reduce the risk of marker artifacts during transfer, a primer-level in silico screen across 19 wheat assemblies predicted no wheat amplicon for the *fcp1070* (Supplementary Methods S1; Supplementary Tables S5-S6), demonstrating R-specific behavior in wheat × triticale progeny. If, as our preliminary data indicate, the 5R segment also retains proximity to *Xct1* (data not shown), breeders could co-deploy nematode and bacterial resistance in a single introgression. What remains uncertain are the local fine-scale recombination rate in this interval and any small structural differences that could dampen crossovers; these caveats are typical of alien transfers but are managed in practice by selecting recombinant breakpoints with flanking markers (Li et al. 2016; Moskal et al. 2021). In a broader context, this work exemplifies how strategic mining of the wheat secondary gene pool, coupled with contemporary genomics, can diversify the resistance landscape beyond the narrow set of loci currently available in bread wheat.

In conclusion, we identified and mapped a novel QTL, *QRlnn.ndsu-5R*, associated with resistance to *P. neglectus* in triticale. To our knowledge, no RLN-resistance locus had previously been mapped in this crop. The tightly linked KASP assay we provided should enable efficient marker-assisted introgression of the 5R resistance allele into triticale and wheat breeding programs, thereby reducing the time and cost associated with phenotypic screening of nematodes. By incorporating rye-derived defense genetics into the cereal improvement pipeline, these results expand the allelic repertoire available for nematode control and contribute to the development of more resilient cultivars.

## Supplementary Information

Below is the link to the electronic supplementary material.

Supplementary file1 (DOCX 19 kb)

Supplementary file2 (XLSX 881 kb)

Supplementary file3 (PDF 6979 kb)

Supplementary file4 (PDF 145 kb)

## Data Availability

The datasets generated during and/or analyzed during the current study are available as supplementary materials and/or from the corresponding author on reasonable request.
