# Supplementary material for: Genetic analysis of a quantitative trait locus associated with resistance to the root-lesion nematode Pratylenchus neglectus in triticale
Source: Theor Appl Genet. 2026 Jan 5;139(1):24. doi: 10.1007/s00122-025-05112-6 (PMC12769958; doi:10.1007/s00122-025-05112-6)
Supplement: Supplementary file 1 — Supplementary file1 (DOCX 19 kb) [file 122_2025_5112_MOESM1_ESM.docx]

Genetic analysis of a quantitative trait locus associated with resistance to the root-lesion nematode, *Pratylenchus neglectus* in triticale, TAG, Gurminder Singh, Krishna Acharya, Bonventure Mumia, Siddant Ranabhat, Ekta Ojha, Jatinder Singh, Upinder Gill, Sean Walkowiak, Harmeet Singh Chawla, Xuehui Li, Justin Faris, Zhaohui Liu, and Guiping Yan^*^

*Corresponding author: Guiping Yan; [guiping.yan@ndsu.edu](mailto:guiping.yan@ndsu.edu); Department of Plant Pathology, North Dakota State University, Fargo, ND 58102, USA

**Supplementary Methods**

**Supplementary Method S1.** *In silico* assessment of *fcp1070* KASP primer specificity in wheat.

To assess whether the *fcp1070* KASP assay could produce spurious products in *Triticum* *aestivum*, we examined the locus-specific parts of the two allele-specific forward primers and the common reverse primer (with fluorophore tails removed; sequences detailed in Supplementary Table S5 and Table 2) against a representative set of 19 wheat reference assemblies that span various cultivars (listed in Supplementary Table S6). Searches utilized BLAST+ configured for short oligonucleotides; genome assemblies were accessed and cross-referenced through WheatOmics (Ma et al. 2021; http://wheatomics.sdau.edu.cn/). Each primer was tested independently; additionally, potential primer-pair products were evaluated *in silico* by checking if a forward-primer site and a reverse-primer site co-occur on the same sequence in the proper orientation and within a typical KASP amplicon size window. A wheat product was deemed “predicted” only when both conditions were satisfied: (i) the forward primer had a perfect match at its 3’ terminal base on wheat DNA, and (ii) a compatible reverse-primer site was present as described. BLAST hits failing either requirement were excluded from consideration as productive under routine KASP conditions. The allele-specific forward primer for the resistance-linked allele places the discriminating nucleotide at the 3’ end, aligned with standard KASP design practices. Primer-level results are summarized in Supplementary Table S5, and per-assembly outcomes along with genome panel details are provided in Supplementary Table S6.

**References**

Ma S, Wang M, Wu J, Guo W, Chen Y, Li G, Wang Y, Shi W, Xia G, Fu D, Kang Z, Ni F (2021) WheatOmics: A platform combining multiple omics data to accelerate functional genomics studies in wheat. Mol Plant. 14:1965-1968. <https://doi.org/10.1016/j.molp.2021.10.006>
