# Supplementary figures and images for: Genetic analysis of a quantitative trait locus associated with resistance to the root-lesion nematode Pratylenchus neglectus in triticale
Source: Theor Appl Genet. 2026 Jan 5;139(1):24. doi: 10.1007/s00122-025-05112-6 (PMC12769958; doi:10.1007/s00122-025-05112-6)

## 1A

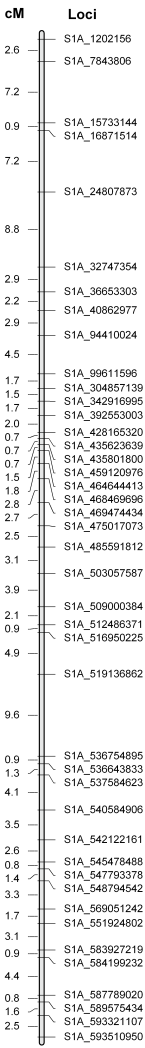

## 2A

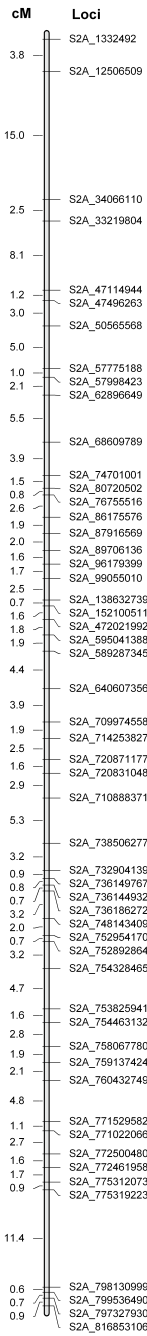

## 3A

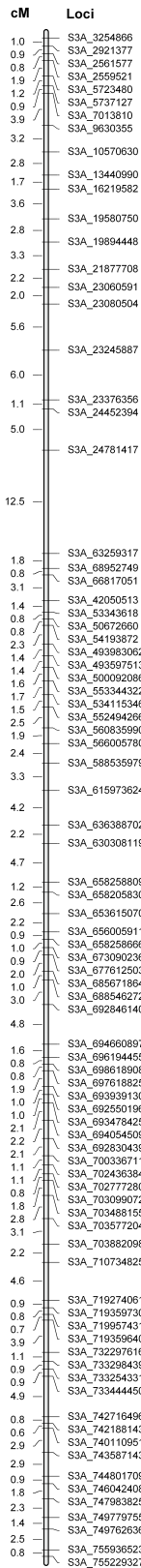

## 4A

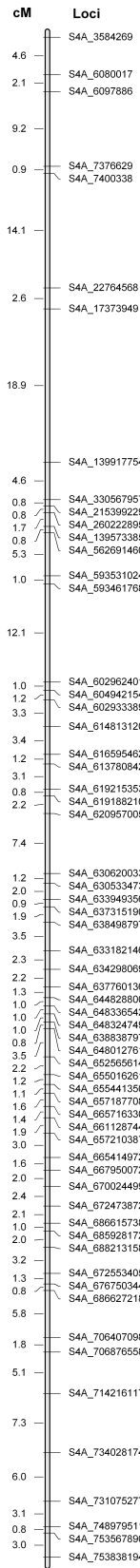

## 5A

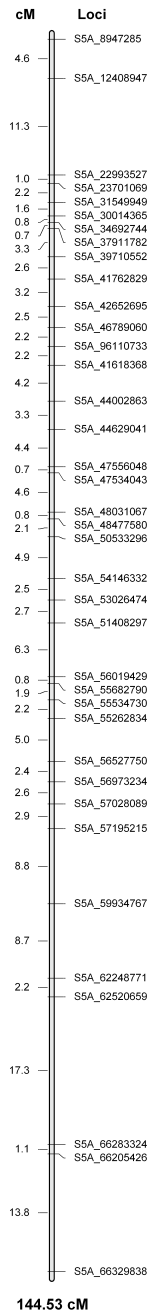

## 6A

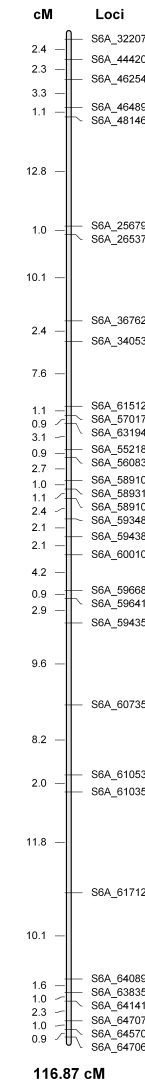

## 7A

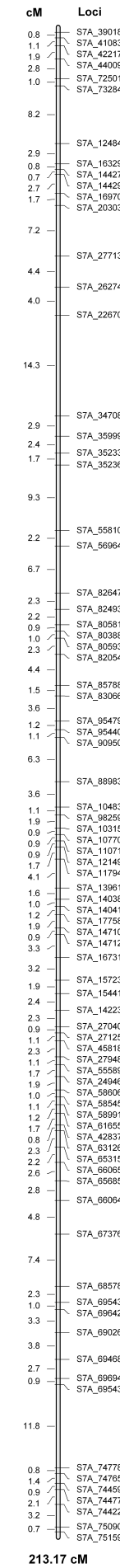

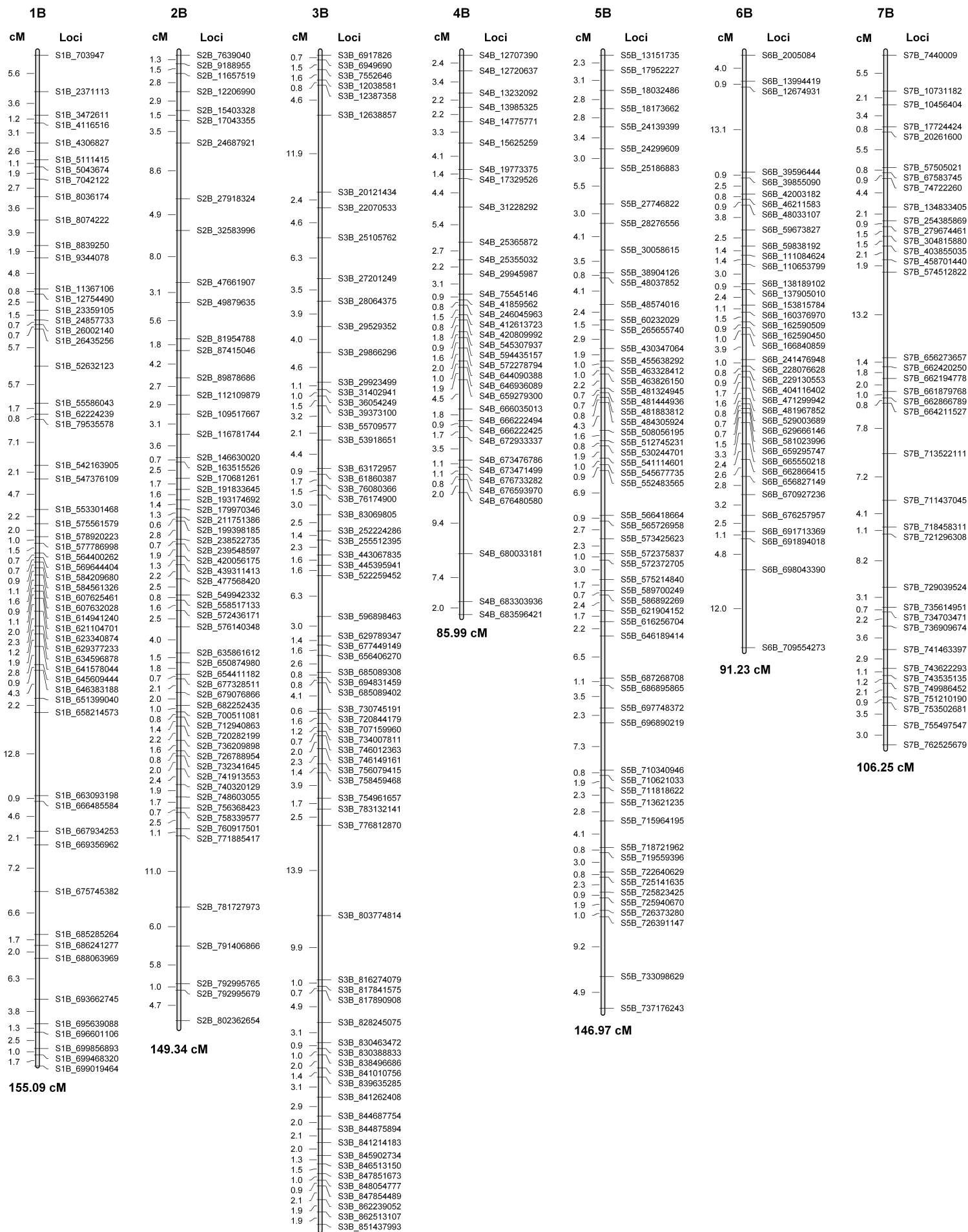

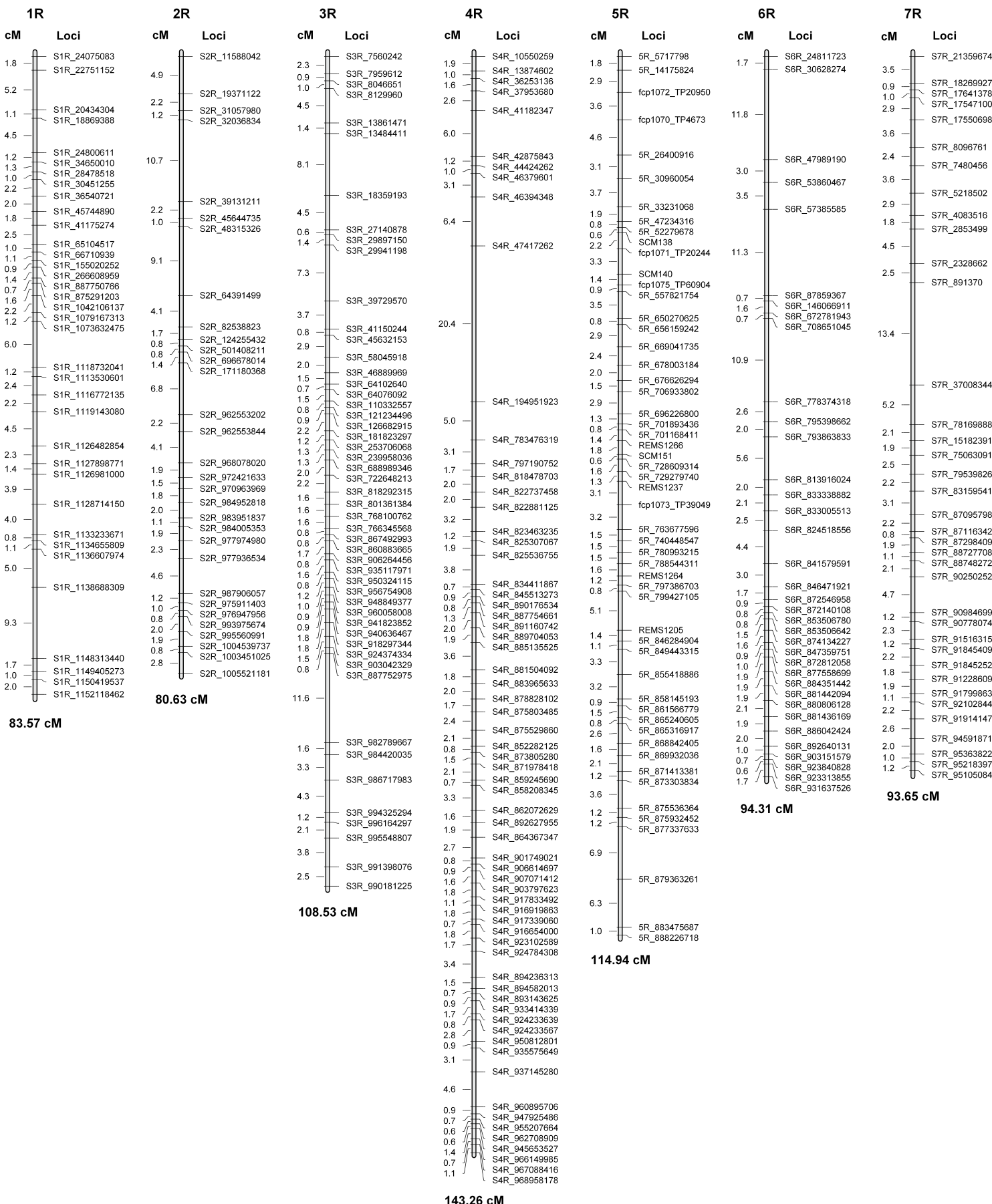

Supplement: Supplementary file 3 — Supplementary file3 (PDF 6979 kb) [file 122_2025_5112_MOESM3_ESM.pdf]

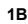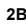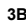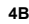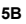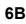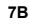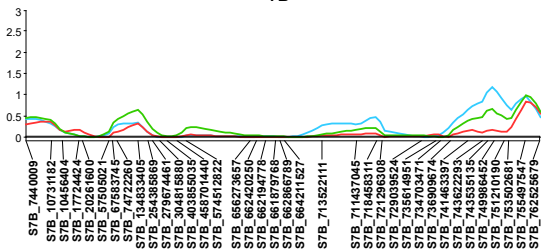

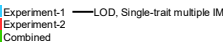

Supplement: Supplementary file 4 — Supplementary file4 (PDF 145 kb) [file 122_2025_5112_MOESM4_ESM.pdf]
